# Osmotic Demyelination Syndrome Following Rapid Correction of Hyponatremia in a Young Woman: A Case Report and Review of Literature

**DOI:** 10.7759/cureus.86452

**Published:** 2025-06-20

**Authors:** Aqsa Akram, Usman Shahbaz Muhammad, Abdulkadir M Ali, Farhan M Rizvi

**Affiliations:** 1 Internal Medicine, Dallah Hospital, Riyadh, SAU; 2 Internal Medicine, Care Medical Hospital, Riyadh, SAU; 3 Diagnostic Radiology, Dallah Hospital, Riyadh, SAU

**Keywords:** central pontine myelinolysis (cpm), demyelination syndrome, extrapontine myelinolysis (epm), locked-in, osmotic demyelination syndrome (ods)

## Abstract

Osmotic demyelination syndrome (ODS) is a rare and devastating neurological condition linked with the rapid correction of serum hyponatremia. We present a case report of a young female patient who developed ODS following an aggressive correction of low serum sodium levels. ODS is characterized by demyelination in the central and extrapontine regions of the brain, resulting in disastrous outcomes. The pathophysiology involves disruption of the blood-brain barrier (BBB) due to a sudden rise in serum sodium, which leads to astrocyte dysfunction secondary to osmotic shift, leading to inflammation, brain edema, and finally demyelination. A rapid rise in the serum sodium levels can overwhelm the brain’s adaptive capacity, ultimately leading to ODS. Our case emphasizes the importance of careful sodium correction; in our patient, the serum sodium levels were raised precipitously, beyond the recommended 8-10 mmol/L limit within its first 24 hours, leading to calamitous neurological consequences. Despite the management of this disastrous condition with plasmapheresis, the patient succumbed to complications. A review of the literature suggests that no definitive treatment of ODS exists; therefore, cautious monitoring and raising the serum sodium levels to prevent ODS is critical. Our case report also highlights the necessity of heedful management of hyponatremia to prevent permanent neurological injury.

## Introduction

Osmotic demyelination syndrome (ODS) is a rare neurological condition first reported by Adams and his team in 1959 in patients who were both undernourished and alcohol dependent [1]. It is characterized by non-inflammatory symmetrical demyelination encompassing the base of the pons (central pontine myelinolysis, or CPM) or outside the pons (extrapontine myelinolysis, or EPM) [2]. The exact mechanism of ODS has not been reported; however, it is proposed to be linked with the rapid correction of low serum sodium levels [3]. The clinical features of the ODS include lethargy, confusion, altered mental status, disorientation, dysarthria, dysphagia, coma, quadriplegia, and pseudobulbar palsy [1,4]. These symptoms develop within two to six days following the rapid correction of serum hyponatremia and are often irreversible [4]. Here, we report a fatal case of ODS that developed after the rapid correction of serum sodium levels in a 29-year-old female with an undiagnosed pituitary adenoma and chronic, asymptomatic hyponatremia.

## Case presentation

**Figure 1:**
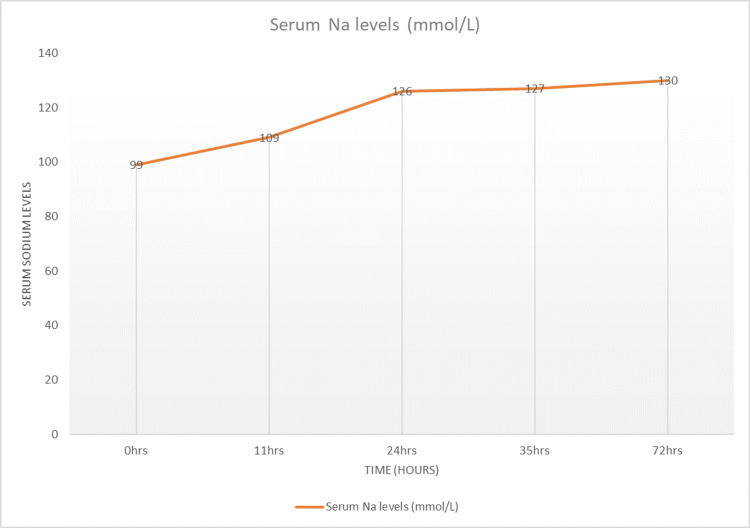
Serum Sodium (Na) Levels During the First 72 Hours of Admission.

A 29-year-old female with no known chronic illness presented to the emergency department complaining of headache, lower abdominal pain, nausea, and non-bilious vomiting associated with extreme fatigue and lethargy lasting for 24 hours. She had a history of irregular menstruation, primary infertility, and endometriosis, and was taking progestin 2 mg. On examination, her vital signs were within normal range; she was conscious and oriented to time, place, and person. At the time of visit, she was ambulatory, and her systemic physical examination was unremarkable. A complete metabolic panel revealed serum sodium level 99 mmol/L, serum potassium 3.1 mmol/L, blood urea nitrogen (BUN) 0.82 mmol/L, serum creatinine 34 umol/L, and blood urea 0.82 mmol/L. Urine analysis showed ketonuria (++), pH 5, urine specific gravity 1.011, and glucose negative. Her serum osmolality was 199.4 mOsm/kg, urine sodium was 16.3 mmol/L, and urine osmolality was 53.1 mOsm/kg. She was initially diagnosed with hypovolemic hyponatremia due to severe vomiting; differential diagnosis included pelvic inflammatory disease. She was admitted to a high-dependency unit and was started on intravenous (IV) 3% hypertonic saline infusion at a rate of 20cc/hr, along with a high-sodium diet. Her serum sodium level and other electrolytes were monitored periodically. Her serum sodium level reached 109 mmol/L after the first 11 hours and 126 mmol/L after 24 hours, increasing to 127 mmol/L after 35 hours and 130 mmol/L within 72 hours (see Figure 1).

**Figure 2:**
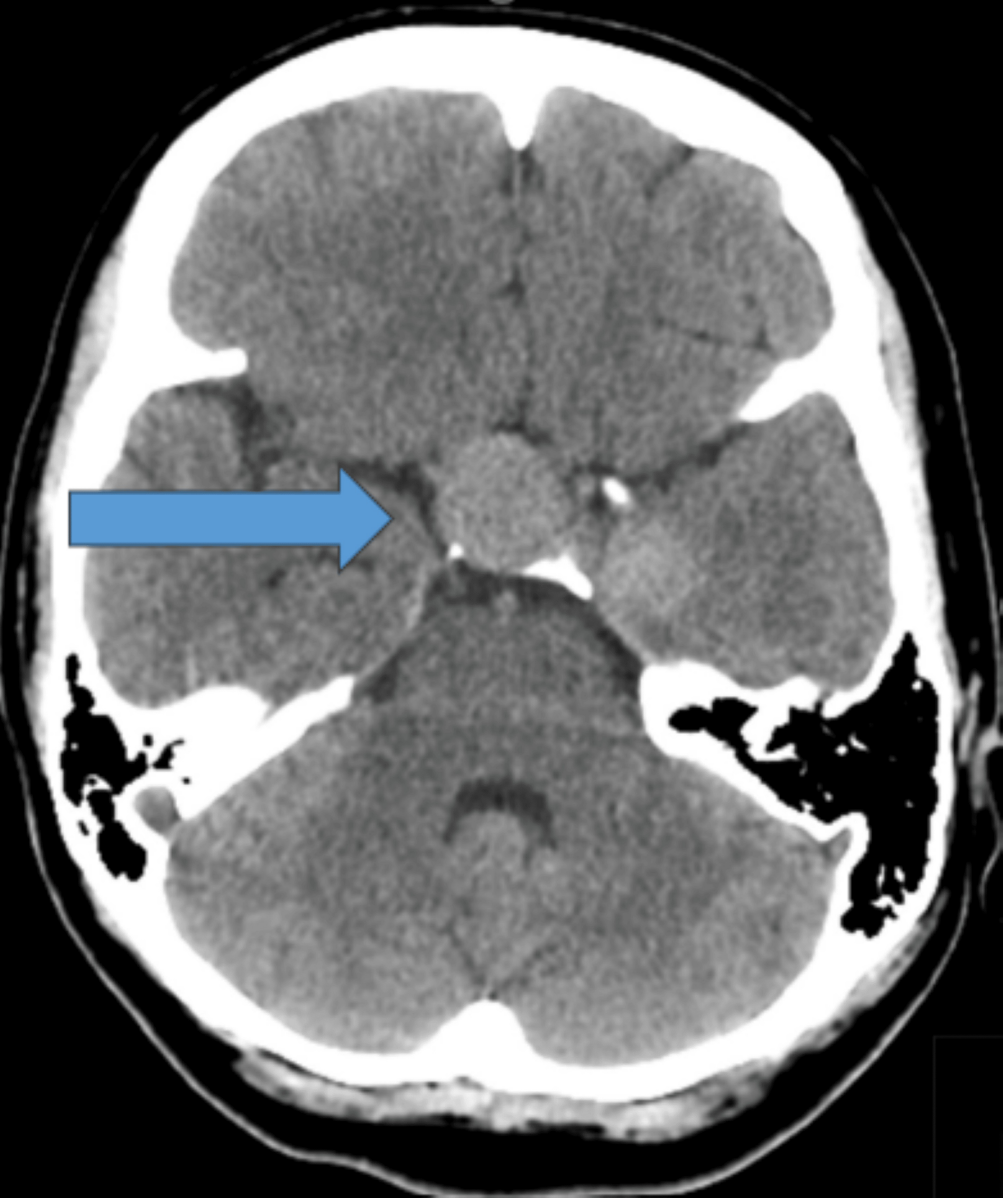
Axial Plane CT Scan Showing Hyperdense Pituitary Fossa (Blue Arrow). CT: computed tomography

Over the first 72 hours, the patient achieved clinical stability and was scheduled for discharge. However, shortly before she was scheduled to leave the hospital, her state of consciousness started to deteriorate. She became drowsy and somnolent, with generalized limb weakness. Shortly afterwards, she experienced recurrent generalized tonic colonic seizures. Neurological examination showed E3M2V1 resulted in a Glasgow Coma Scale (GCS) score of 6/15, bilateral central nystagmus with the rapid phase to the right side, and weak reflexes. She was promptly shifted to an intensive care unit (ICU) and was intubated. She was started on IV phenytoin 1000 mg infusion, along with potassium chloride (KCl) 20 mEq in 500 mL of 5% dextrose in normal saline (D5NS) infused over four hours. She received midazolam 2 mg IV infusion and propofol 30 mg/kg/min IV infusion. An urgent CT scan of her brain revealed a large, well-defined hyperattenuating seller mass extending to the right parasellar region, consistent with pituitary macroadenoma (see Figure 2).

**Figure 3:**
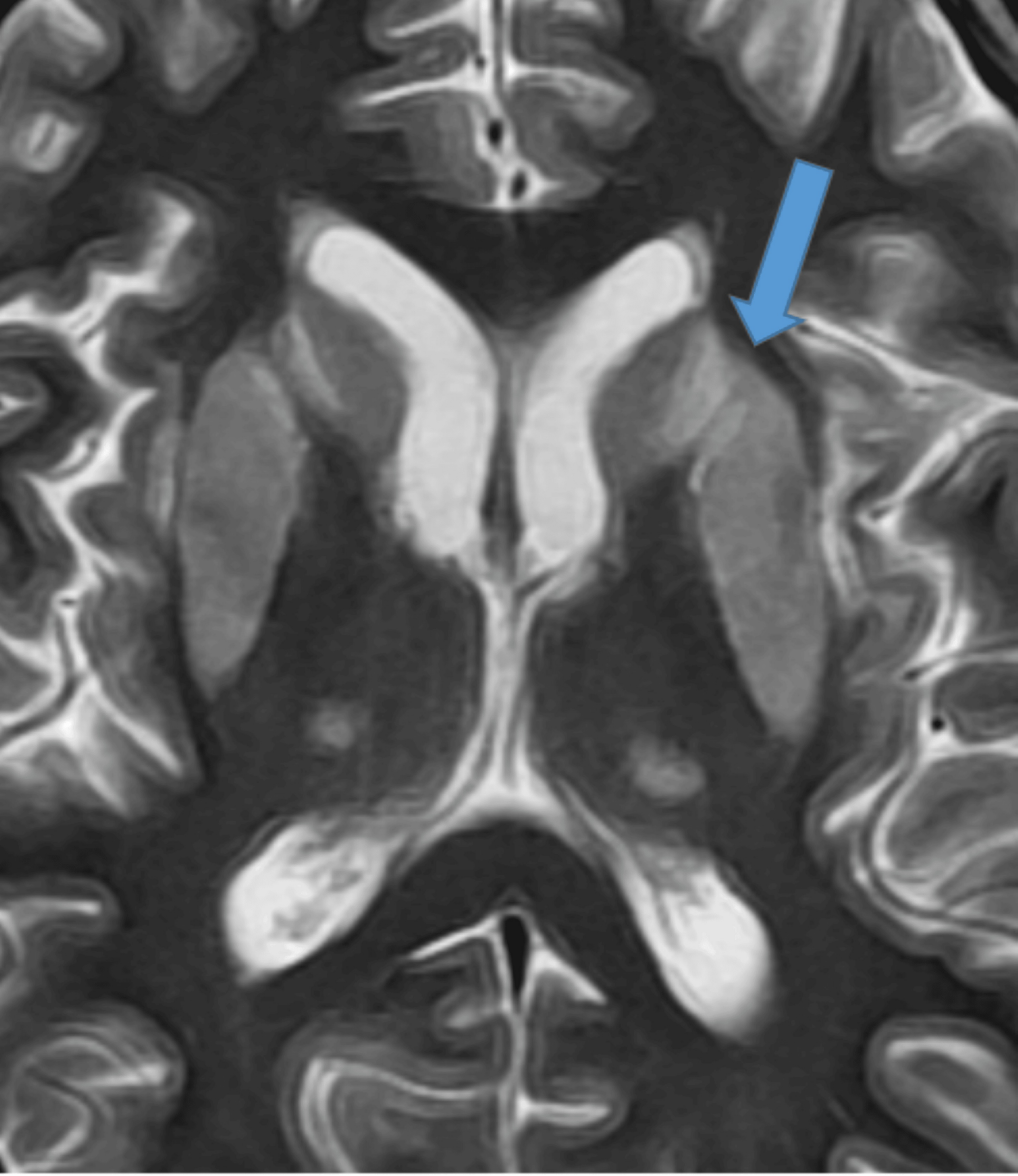
T2-Weighted Axial MRI Sequence Showing Hyperintense Signal (Blue Arrow). MRI: magnetic resonance imaging

**Figure 4:**
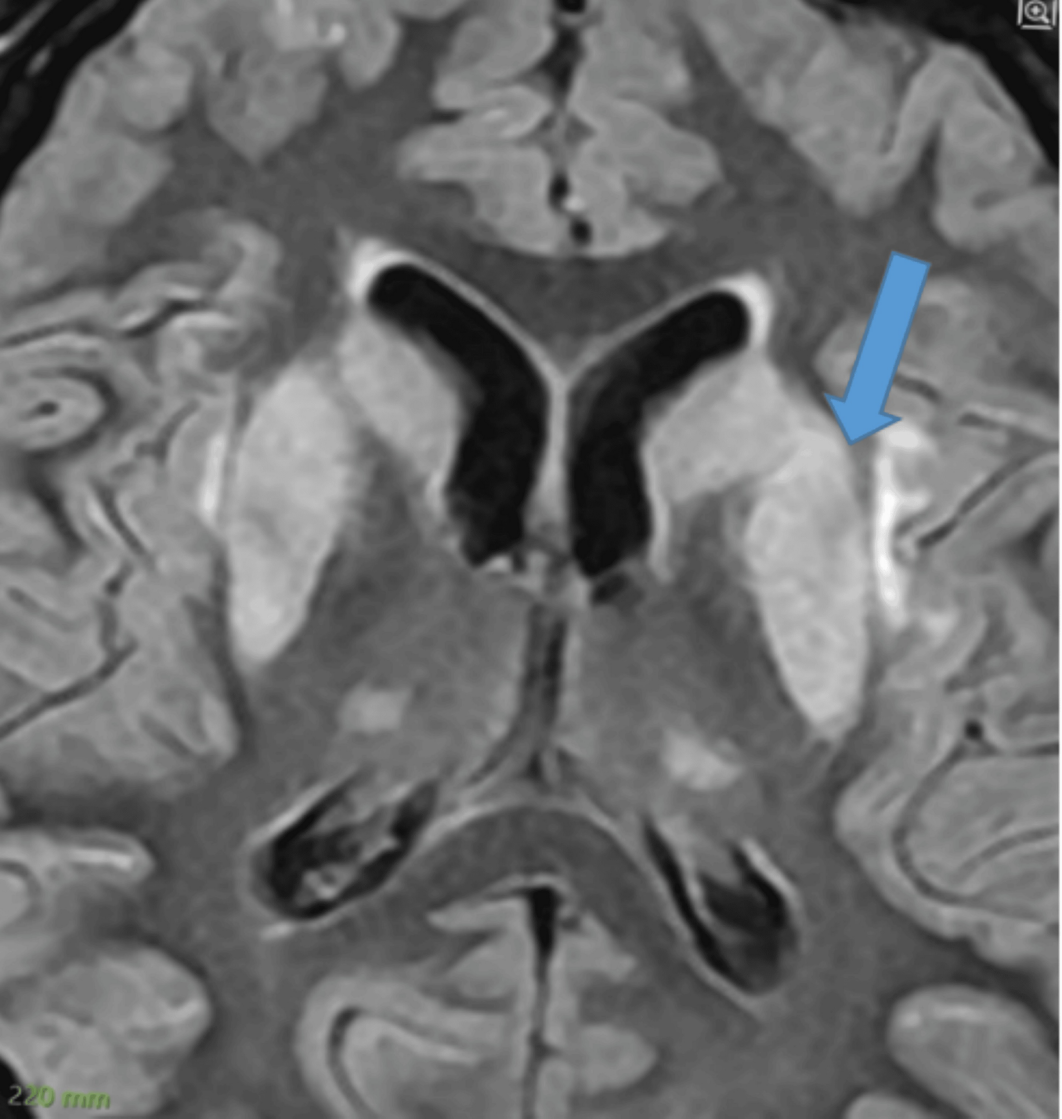
FLAIR Axial MRI Sequence Showing Hyperintense Signal (Blue Arrow). FLAIR: fluid-attenuated inversion recovery; MRI: magnetic resonance imaging

**Figure 5:**
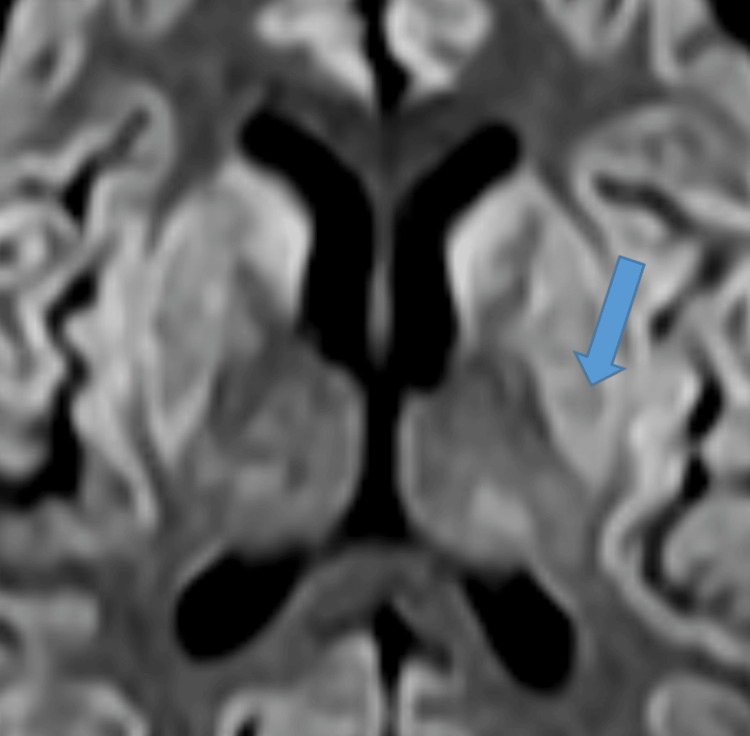
DWI Axial MRI Sequence Showing Hyperintense Signal (Blue Arrow). DWI: diffusion-weighted imaging; MRI: magnetic resonance imaging

**Figure 6:**
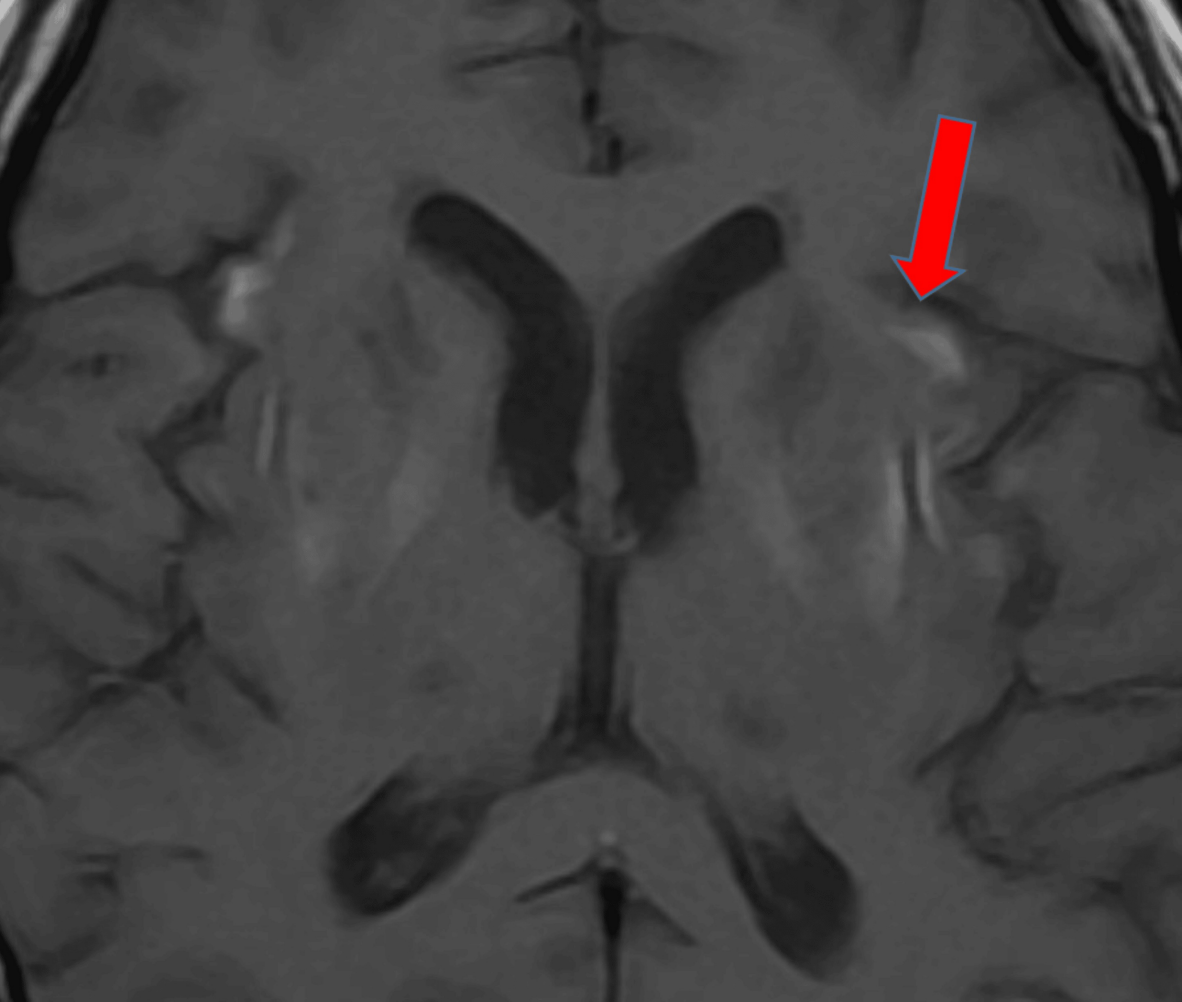
T1 Axial MRI Sequence Showing Hypointense Signal With Patchy Areas of Hyperintensity (Red Arrow), Suggestive of Hemorrhagic Transformation. MRI: magnetic resonance imaging

Following the CT scan, an endocrinological assessment and an MRI were performed. The former revealed adrenocorticotrophic hormone (ACTH) at 11 pg/mL, cortisol at 24.92 µg/dL, free T3 (FT3) at 1.94 pmol/mL, prolactin at 32.20 ng/mL, follicle-stimulating hormone (FSH) at 2.94 mIU/mL, and luteinizing hormone (LH) at 0.04 mIU/mL. The brain MRI showed an almost bilateral symmetrical laminar pattern of cortical subcortical involvement of the cerebral hemisphere, caudate head and lentiform nuclei, external capsule, lateral part of thalami, and central pons, suggestive of CPM and EPM (Figures 3-5). A T1-weighted axial image demonstrates hypointense signal with patchy areas of hyperintensity, suggestive of hemorrhagic transformation within the region of myelinolysis involving the cortical rim and basal ganglia (Figure 6, red arrow).

**Figure 7:**
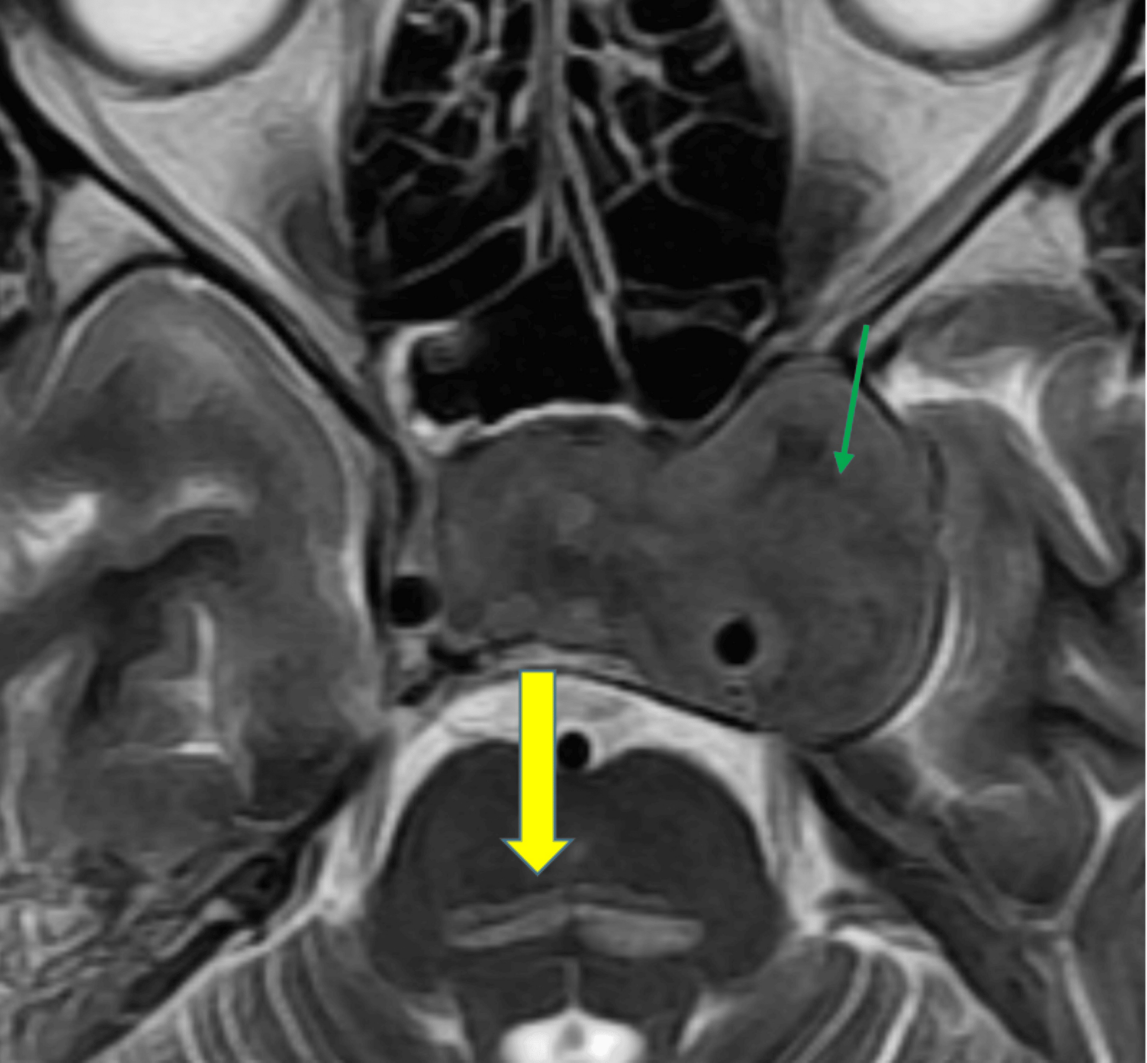
T2 Axial MRI Sequence Showing Pontine Myelinolysis (Yellow Arrow) and a Large Pituitary Macroadenoma (Green Arrow). MRI: magnetic resonance imaging

**Figure 8:**
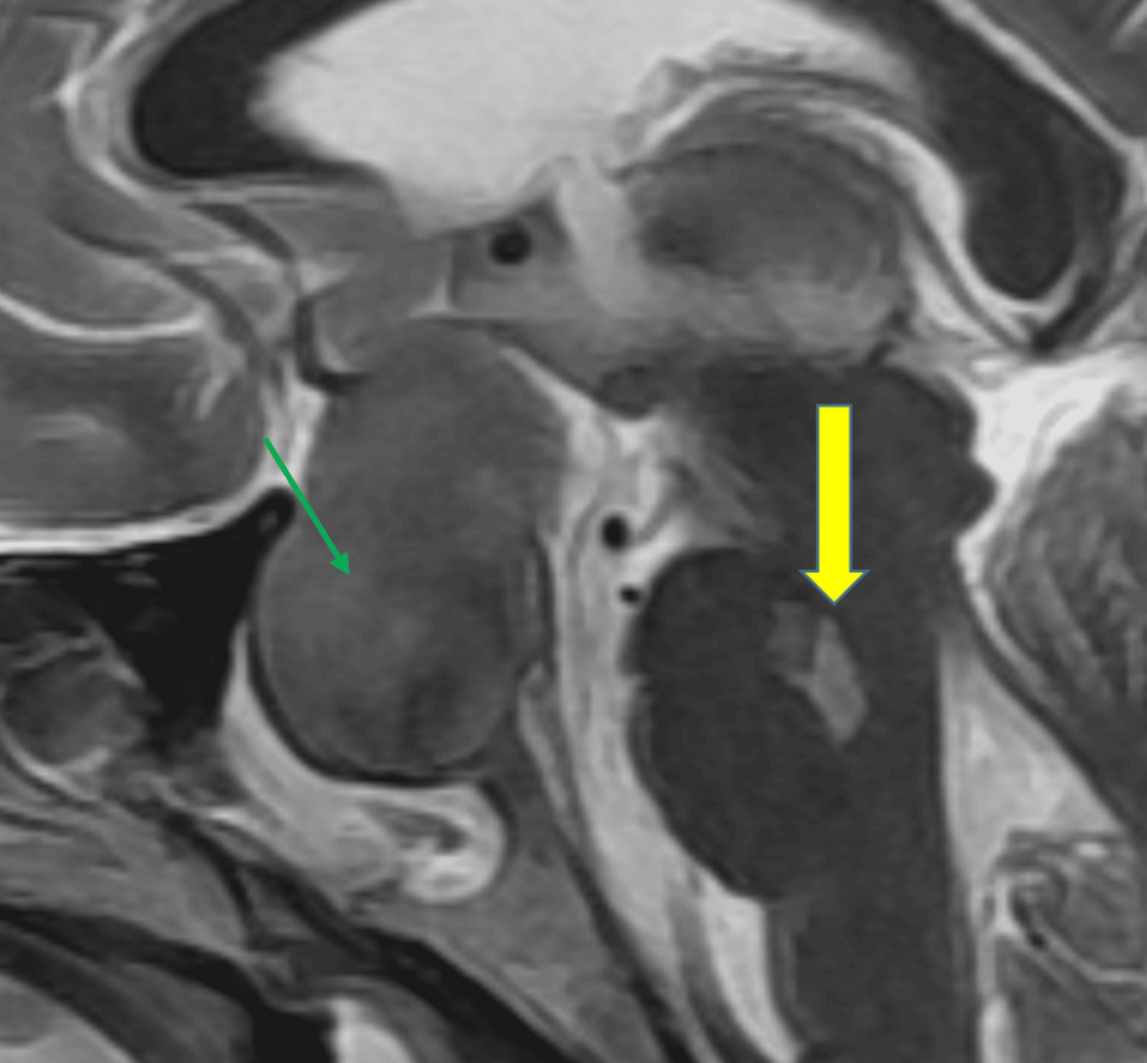
T2 Sagittal MRI Sequence Showing Pontine Myelinolysis (Yellow Arrow) and a Large Pituitary Macroadenoma (Green Arrow) MRI: magnetic resonance imaging

Further imaging (Figures 7-8) indicated pontine myelinolysis (yellow arrow) and a large pituitary macroadenoma with suprasellar and left parasellar extension encircling the left internal carotid artery (ICA) (green arrow), which was identified as the cause of hyponatremia.

In light of this diagnostic imaging, the patient was given dexamethasone 8 mg IV infusion and cabergoline 0.5 mg IV infusion daily for one month by the endocrinologist. The patient was kept on a mechanical ventilator; her GCS was 6/15, and she was weaned off sedation. Ten days later, her consciousness level had shown no improvement; she demonstrated weak brainstem reflexes. A follow-up MRI revealed progression of the demyelination process.

An electroencephalogram showed asymmetric periodic slow episodic activity consistent with severe encephalopathy and left focal lesion. After 30 days, the patient’s GCS score was 7/15; pupils were equal and reactive, with positive clonus and brisk reflexes. After 40 days in the ICU, she was still on the mechanical ventilator, with improving GCS scores (10/15) and good brainstem reflexes. A trial of weaning the patient off the ventilator was attempted, but proved unsuccessful due to pseudobulbar palsy; she was reintubated due to desaturation. Neurological examination showed E3M2V1, resulting in a GCS score of 6/15, along with weak neurological reflexes, bilateral central nystagmus with the rapid phase to the right side, pseudobulbar palsy, and dysarthria. Throughout this period, the patient continued to receive all supportive care indicated in patients with ODS, including chest physiotherapy, nasogastric tube (NGT) feeding, and frequent suctions to prevent any complications associated with aspiration. She had a total of five sessions of plasmapheresis every two days; after the initial two sessions, the patient showed some improvement in her movements (bilateral lower limb 3/5, bilateral upper limbs 2/5). However, after completing all sessions, the therapy did not show any significant benefit. Post 83 days, she developed sudden massive bleeding from the tracheostomy tube due to tracheostomy block by clot and became hypotensive; afterwards, she experienced cardiopulmonary arrest and was pronounced dead after 15 minutes of unsuccessful cardiopulmonary resuscitation, as per advanced cardiac life support protocol.

## Discussion

Literature review

We report here a rare case of ODS in a young female patient who had fatal complications following the rapid correction of the serum sodium levels. To understand the pathophysiology of ODS, we need to explore the basics of osmolality in the human body, as explored in the literature.

Physiology of Osmosis and Water Movement

Human cells are made up of a semipermeable phospholipid bilayer membrane, which allows the filtration of some substances and leads to the process called osmosis [5]. During osmosis, two compartments separated by a semipermeable membrane are filled with a solute and solvents; the main driving force of these solvents is the concentration of osmotically active solute in one compartment, which makes up for the osmotic pressure in the other [5]. Water moves freely across these compartments and will follow the compartment where osmotic pressure is high to equalize the osmotic pressure in both compartments (within and outside the cells) [5]. This movement of solute and solvent is crucial to maintaining normal osmotic pressure within cells. The main solute in extracellular compartments, responsible for the determination of osmolarity, is sodium. Changes in the sodium concentration in extracellular fluid (ECF) will directly affect the osmolality of the cells [5].

Hyponatremia is the most common electrolyte disturbance, seen almost every day in emergency departments; it accounts for up to 10% of hospital admissions [6]. European guidelines define hyponatremia as a serum sodium level below 135 mmol/L [6]. The degree of hyponatremia can be categorized based on the serum concentration of sodium into three categories: mild (130-135 mmol/L), moderate (125-129 mmol/L), and severe (less than 125 mmol/L) [6]. Clinically, it is classified as either acute or chronic based on the duration of the lowered serum sodium levels; a drop in serum sodium lasting less than 48 hours is classified as acute hyponatremia, whereas a drop in serum sodium persisting for more than 48 hours is classified as chronic hyponatremia [5].

Before attempting management of hyponatremia, it is crucial to consider the ECF status, which can guide us to the underlying possible etiology of the condition [7]. One method of immediate clinical evaluation entails observing patients presenting to the emergency room for signs of hypovolemic ECF status, including decreased skin turgor, dry mucous membranes, delayed capillary refill time, tachycardia, and orthostatic hypotension [7].

To manage and approach any patient with hyponatremia, it is crucial to take a step back into the etiology of the underlying electrolyte abnormality. The most common attributable risk factors for hyponatremia include cerebral pathologies, such as intracranial injuries; surgery; chronic use of diuretics, especially thiazide diuretics; heart failure; liver cirrhosis; syndrome of inappropriate antidiuretic hormone, polydipsia; certain antipsychotic medications; beer potomania; and endocrinological pathologies such as hypothyroidism and adrenal insufficiency [6,8]. Clinical manifestation of hyponatremia can be asymptomatic or entail milder symptoms such as lethargy, confusion, headache, vomiting, abdominal pain, and falls [9]. Regardless of the etiology and symptoms, hyponatremia must be considered as a medical emergency and must be treated to prevent morbidity and mortality [9].

Clinical Management and Consequences of Correction of Serum Sodium Levels

ODS is a rare but devastating neurological condition that has been linked with rapid correction of hyponatremia, particularly when serum sodium levels are elevated precipitously. Although the exact mechanism of ODS is poorly understood, it is believed that a steep rise in the serum sodium levels increases the ECF’s osmolarity levels, which, in turn, results in disruption of the blood-brain barrier (BBB), releasing the myelin toxins, resulting in brain cytotoxicity and subsequent demyelination [3,4,10]. Risk factors for ODS are secondary to other chronic conditions, most notably alcoholism. Patients with diabetes who develop hyperosmolar hyperglycemia are at risk of hyponatremia, which will eventually lead to ODS, hepatic failure, high serum ammonium levels, and hypoxia [11]. This syndrome affects both the central and peripheral pontine regions.

CPM primarily involves the central pontine, while EPM classically affects the thalamus, basal ganglia, or subcortical white matter. The distinction among the lesions results in varying clinical presentations, with symptoms such as dystonia, dysarthria, confusion, altered mentation, and even death often delayed up to several days following the rapid elevations in the serum sodium levels [3]. The role of astrocytes in brain homeostasis cannot be overstated; these glial cells are critical in maintaining the intracellular volume balance in response to changes in ECF osmolarity, as these cells lose their ability to maintain volume homeostasis as ODS unfolds [12]. Within the first 24 to 48 hours of osmotic dysregulation, astrocytes protect neurons from osmotic stress by transferring taurine to adjacent cells and by losing organic osmolytes. However, in the case of rapid hyponatremia correction, these cells are unable to accommodate the speed of the change, leading to a cascade of harmful effects and ending up disrupting the BBB [9]. As astrocytes lose their integrity and function, pro-inflammatory cytokines, in combination with complement activation, create a hostile environment that exacerbates neural damage and ultimately leads to demyelination, impairs neuronal communication, and exacerbates neurological dysfunction [2,9].

When considering treatment for hyponatremia, it is also important to understand the possible underlying causes of this electrolyte disturbance, as addressing the underlying abnormality leads to normalization of serum sodium levels. The correction of chronic hyponatremia should be approached cautiously over a period of days. Patients with chronic hyponatremia typically show no signs of brain edema or alteration in brain function, as they have fully adapted functionality. Raising sodium levels should be done gradually, with close monitoring of serum sodium levels [5]. In our case, the patient presented with no signs of acute altered brain function and no changes in functionality, yet the serum sodium levels were raised aggressively in treatment, as if in the case of acute hyponatremia, which led to a devastating outcome. It is still not well-reported whether treating mild hyponatremia will have any clinical added benefits for the overall outcome or not [5]. Overcorrection of serum sodium levels is defined as raising serum sodium levels greater than 8-10 mmol/L within the first 24 hours [6]. Several studies have recommended the correction rate of serum sodium regardless of chronicity, a rapid rise of 4-6 mEq/L or less than 10 mmol/L in any 24-hour period [8,13]. Some studies have even recommended that the rate should not exceed 8 mmol/L per 24 hours to prevent this devastating neurological outcome [2]. In our case, the initial rise in serum sodium level was beyond 10 mmol/L, which put this patient’s brain cells at risk of rapid osmotic shift; accordingly, the astrocytes failed to maintain their integrity, resulting in inflammation and eventually demyelination. Therefore, we must conclude that the rate of correction of serum sodium, which surpassed 12 mmol/L in the first 24 hours and totaled 20 mmol/L within 48 hours, was the main cause of the clinical symptoms of CPM/EPM [2]. Clinical manifestation of lesions in the central pontine region encompassed seizures and altered mentation, which is the initial phase of the CPM biphasic course, mainly encephalopathy. The later clinical phase consists of oculomotor abnormalities, dysarthria, dysphagia, quadriparesis (also called locked-in syndrome), behavioral symptoms, and personality disturbance; movement abnormalities were also reported in patients with lesions involving EPM [14]. The diagnosis of ODS can be confirmed with a gold standard diagnostic modality by a brain MRI, presenting with hypointense T1-weighted and hyperintense T2-weighted signal [3,15]. In our patient, the MRI confirmed classic features of myelinolysis in the central pons and extrapontine regions, as highlighted in Figures 3-5.

Some studies have also reported demyelination cases even when an appropriate correction rate was implemented; these cases were later explained by other factors, such as the duration of hyponatremia and serum concentration at the time of diagnosis, as well as certain risk factors such as low serum potassium at the initial presentation, low body mass index, history of undernourishment, and chronic alcohol use disorder [6,8]. Once the diagnosis of ODS is established, there are no proven modalities to treat the condition or reverse the osmotic dysfunction. A few studies on animals have reported that lowering the high serum sodium rapidly after a steep rise in serum sodium could have preventive benefits [4,6,16]. Apart from supportive therapies, some studies have mentioned the use of plasmapheresis, dexamethasone, thyrotropic-releasing hormone, and immunoglobulin therapy [16]. Plasmapheresis was used to remove the extracorporeal cytotoxic fluid in combination with other treatment modalities; the outcome after these therapies was not particularly promising, however, and results were variable in different studies [8,16]. We tried five sessions of plasmapheresis in our patient once the diagnosis of ODS was established; after the initial two sessions, she showed some clinical improvement in overall muscle tone, but after five sessions, the patient developed massive bleeding from her tracheostomy tube and eventually developed hypotensive shock and cardiopulmonary arrest. As previously reported in different studies, even when efforts to prevent the development of ODS fail, patients can survive if complications such as pulmonary embolism, deep venous thrombosis, and aspiration pneumonia are avoided [8]. The overall outcome is still catastrophic, with high mortality rates, and despite all efforts, even if the patient survives, the chances of irreversible neurological deficits are high.

## Conclusions

This case report highlights the critical importance of managing serum sodium levels in cases of hyponatremia. We present a fatal case of ODS, a severe neurological condition that causes irreversible neurological damage and can ultimately lead to death. A thorough review of the literature led us to several key conclusions for treating electrolyte disturbance in the emergency setting. It is essential to consider several factors, such as the underlying etiology, duration, and the body’s compensatory mechanisms, particularly in cases of chronic hyponatremia. These factors play a crucial role in guiding the decision-making process for initial fluid management. According to various published guidelines, the initial 24 hours of treatment are critical in preventing ODS. We emphasize that clinicians should raise serum sodium levels gradually and cautiously, monitoring serum sodium levels to avoid such a disastrous outcome, as there is currently no effective treatment for ODS.

## References

[1] Adams RD, Victor M, Mancall EL (1959). Central pontine myelinolysis: a hitherto undescribed disease occurring in alcoholic and malnourished patients. AMA Arch Neurol Psychiatry.

[2] Zhao P, Zong X, Wang X, Zhang Y (2012). Extrapontine myelinolysis of osmotic demyelination syndrome in a case of postoperative suprasellar arachnoid cyst. Case Rep Med.

[3] Fang LJ, Xu MW, Zhou JY, Pan ZJ (2020). Extrapontine myelinolysis caused by rapid correction of pituitrin-induced severe hyponatremia: a case report. World J Clin Cases.

[4] Kumon S, Usui R, Kuzuhara S, Nitta K, Koike M (2017). The improvement of the outcome of osmotic demyelination syndrome by plasma exchange. Intern Med.

[5] Kengne FG (2023). Adaptation of the brain to hyponatremia and its clinical implications. J Clin Med.

[6] Lindner G, Schwarz C, Haidinger M, Ravioli S (2022). Hyponatremia in the emergency department. Am J Emerg Med.

[7] Lawless SJ, Thompson C, Garrahy A (2022). The management of acute and chronic hyponatraemia. Ther Adv Endocrinol Metab.

[8] Zhou Y, Zhu Y, Wang W, Xing B (2016). Preoperative extrapontine myelinolysis with good outcome in a patient with pituitary adenoma. J Korean Neurosurg Soc.

[9] Kheetan M, Ogu I, Shapiro JI, Khitan ZJ (2021). Acute and chronic hyponatremia. Front Med (Lausanne).

[10] Gayathri DK, Dhayalen K, Chia YK, Fung YK (2019). Plasmapheresis to treat osmotic demyelination syndrome from overly rapid plasma sodium correction. Med J Malaysia.

[11] George JC, Zafar W, Bucaloiu ID, Chang AR (2018). Risk factors and outcomes of rapid correction of severe hyponatremia. Clin J Am Soc Nephrol.

[12] Tandukar S, Sterns RH, Rondon-Berrios H (2021). Osmotic demyelination syndrome following correction of hyponatremia by ≤10 mEq/L per day. Kidney360.

[13] Tzamaloukas AH, Malhotra D, Rosen BH, Raj DS, Murata GH, Shapiro JI (2013). Principles of management of severe hyponatremia. J Am Heart Assoc.

[14] Sharma C, Kumawat BL, Panchal M, Shah M (2017). Osmotic demyelination syndrome in type 1 diabetes in the absence of dyselectrolytaemia: an overlooked complication?. BMJ Case Rep.

[15] Wijesundara D, Senanayake B (2022). Plasmapheresis for extrapontine myelinolysis: a case series and a literature review. Case Rep Neurol.

[16] Wijayabandara M, Appuhamy S, Weerathunga P, Chang T (2021). Effective treatment of osmotic demyelination syndrome with plasmapheresis: a case report and review of the literature. J Med Case Rep.

